# Imine Deaminase Activity and Conformational Stability of UK114, the Mammalian Member of the Rid Protein Family Active in Amino Acid Metabolism

**DOI:** 10.3390/ijms19040945

**Published:** 2018-03-22

**Authors:** Genny Degani, Alberto Barbiroli, Luca Regazzoni, Laura Popolo, Maria Antonietta Vanoni

**Affiliations:** 1 Dipartimento di Bioscienze, Università degli Studi di Milano, 20133 Milano, Italy; genny.degani@unimi.it; 2 Dipartimento di Scienze per gli Alimenti, la Nutrizione e l’Ambiente, Università degli Studi di Milano, 20133 Milano, Italy; alberto.barbiroli@unimi.it; 3 Dipartimento di Scienze Farmaceutiche, Università degli Studi di Milano, 20133 Milano, Italy; luca.regazzoni@unimi.it

**Keywords:** UK114, Rid family, metabolic damage, thermal stability, 2-aminoacrylate, imino acids, D-amino acid oxidase, l-amino acid oxidase

## Abstract

Reactive intermediate deaminase (Rid) protein family is a recently discovered group of enzymes that is conserved in all domains of life and is proposed to play a role in the detoxification of reactive enamines/imines. UK114, the mammalian member of RidA subfamily, was identified in the early 90s as a component of perchloric acid-soluble extracts from goat liver and exhibited immunomodulatory properties. Multiple activities were attributed to this protein, but its function is still unclear. This work addressed the question of whether UK114 is a Rid enzyme. Biochemical analyses demonstrated that UK114 hydrolyzes α-imino acids generated by l- or d-amino acid oxidases with a preference for those deriving from Ala > Leu = l-Met > l-Gln, whereas it was poorly active on l-Phe and l-His. Circular Dichroism (CD) analyses of UK114 conformational stability highlighted its remarkable resistance to thermal unfolding, even at high urea concentrations. The half-life of heat inactivation at 95 °C, measured from CD and activity data, was about 3.5 h. The unusual conformational stability of UK114 could be relevant in the frame of a future evaluation of its immunogenic properties. In conclusion, mammalian UK114 proteins are RidA enzymes that may play an important role in metabolism homeostasis also in these organisms.

## 1. Introduction

**Figure 1:**
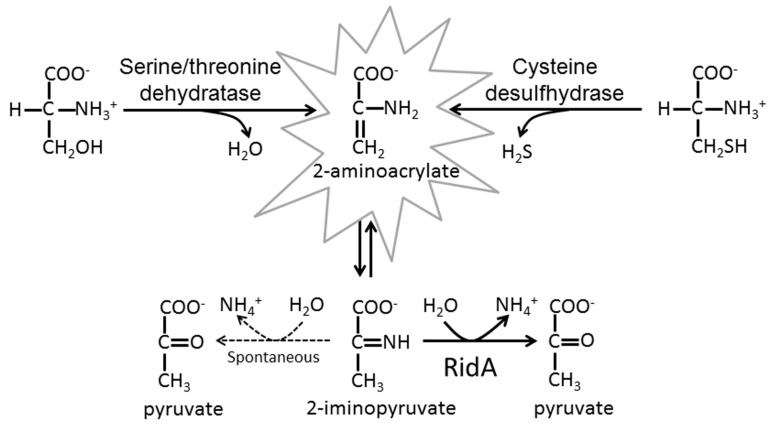
Proposed detoxifying function of the Reactive intermediate deaminase (Rid) proteins. The figure summarizes the reactions catalyzed by the indicated pyridoxal phosphate (PLP)-dependent enzymes leading to the formation of the toxic compound 2-aminoacrylate (2AA) that tautomerizes to 2-iminopropionate (iminopyruvate), which is hydrolyzed to pyruvate and ammonia non-enzymatically following formation of a 2-carbinolamine intermediate (*left branch*, dashed arrows ) or by a RidA protein (*right branch*).

UK114 is the mammalian member of a large family of proteins that are widely distributed in all kingdoms of life and is referred to as the YigF/YER057c/UK114 protein family. The enzymatic activity and the biochemical role of certain members, none of mammalian origin, have been recently elucidated [1,2]. These proteins are involved in metabolic homeostasis, and, in particular, in removing metabolic intermediates that could cause damages to cellular components [3,4]. One newly identified toxic intermediates is 2-aminoacrylate (2AA), which is generated by pyridoxal phosphate (PLP)-dependent serine/threonine dehydratases, with serine as the substrate, or by cysteine desulfhydrases (see scheme in Figure 1) as shown by elegant studies with *Salmonella enterica* that were performed both in vitro and in vivo [2,3,5,6]. By forming adducts with PLP itself and active site residues, 2AA that is produced from serine or cysteine dehydration can inactivate other PLP-dependent enzymes such as alanine racemase (Alr) [7], serine hydroxymethyltransferase (GlyA) [5], branched chain amino acid transaminase B (IlvE) [3], and aspartate aminotransferase [8], but it may also modify other (as yet unknown) physiological targets [2,3,4,9]. Enamines tautomerize to imines, very reactive and labile compounds as well [10], which hydrolyze to the corresponding keto acids and ammonia at a relatively slow rate (half-life of the imine derived from l-Leu oxidation: 6 s at pH 7.7 and 21 s at pH 8.7 [11]; 4 min for the threonine-derived enamine intermediates at pH 9.7 and 25 °C [12], and 1.5 s for the 2-aminoacrylate/iminopyruvate intermediate generated by tryptophanase at pH 8.0 and 30 °C [13], as quoted in [1]).

The complex phenotypes generated by ablation of RidA genes demonstrate the importance of the protein products in buffering 2AA stress [9,14,15,16]. It is remarkable that in some bacteria species lacking RidA, even motility is defective, whereas plants that are lacking RidA activity exhibit reduced root growth [14,17].

The selective pressure imposed by the need to prevent metabolic damage by reactive enamines/imines may explain the conservation of Rid activity from early evolution [6].

In vitro assays have shown how some members of the YigF/YER057c/UK114 protein family accelerate the hydrolysis of the enamine/imine that is generated by Ser/Thr dehydratase from serine, Cys desulfhydrase from cysteine and the imine released from l-amino acid oxidases and different amino acids [1,6,15]. After these discoveries, the YigF/YER057c/UK114 protein family was renamed Rid (Reactive intermediate deaminase) family [6]. The Rid family currently comprises eight subfamilies, RidA and Rid1 to Rid7 [6]. The RidA subfamily includes members from Eukaryotes and Archaea, and is also widely distributed among Eubacteria species, with the best characterized member being RidA from *S. enterica* [4]. The other seven subfamilies (Rid1 to Rid7) are unique to prokaryotes. Interestingly, some bacteria may contain up to 11 different Rid proteins. Specific signature patterns identify each subfamily [6].

Several crystal structures of RidA proteins have been determined long before the discovery of their enzymatic activity [18,19,20]. RidA proteins are homotrimers with the cleft between two adjacent monomers harbouring the active site.

The archetype of the eukaryotic RidA family, and the first mammalian member to be identified, was UK114 originally detected as a component of a perchloric acid-soluble extract from *Capra hircus* (goat) liver. Because of its immunomodulatory properties and its ability to elicit cytotoxic antibodies against some solid tumors, UK114 was described as a tumor-associated antigen [21,22,23,24,25,26,27,28,29,30].

The human homolog of UK114 is encoded by a single gene, annotated as *HRSP12*, localized on chromosome 8 and organized in six exons (http://genome.ucsc.edu/). Previous studies and RNA Seq analyses revealed that—in the adult—the expression of *HRSP12* is high in hepatocytes, lower in renal distal tubular epithelial cell and weak in all of the other tissues. Moreover, *HRSP12* is up-regulated during differentiation and in proliferating cells, while it is down-regulated in tumors [31,32].

Contrasting results concern the intracellular localization of RidA proteins in eukaryotes. Yeast genomes contain two paralogs encoding RidA proteins. In particular, *Saccharomyces cerevisiae* YE0057cR/*HMF1* codes for a cytosolic protein dispensable for viability, whereas its paralog, YIL051c/*MMF1*, encodes a mitochondrial matrix protein that is essential for isoleucine biosynthesis and mitochondria stability [33,34], as further confirmed and clarified in a recently published work [35]. Rat and human homologs of UK114 were localized in the cytoplasm, in the peroxisomal matrix, and, occasionally, in the nucleus of differentiating cells, whereas no evidence of import in the mitochondrion was reported [31,36]. Mammalian UK114 homologs are *N*-acetylated on the second amino acid (a serine residue) [21,29].

Interest on UK114 has also been recently stimulated by the identification of Der f 34, which is a major allergen of house dust mite (*Dermatophagoides farinae*). This homolog of UK114 is endowed with RidA activity [37]. Der f 34 and a component of the spores of the fungus *Aspergillus fumigatus* cross-react with IgE from patients allergic to indoor allergens, suggesting that members of the RidA protein family may represent important pan-allergens that are conserved across different organisms [37].

Although enamine/imine hydrolase activity has been reported in some assays [2,3,33], to our knowledge, an in depth biochemical characterization of a mammalian RidA is still lacking. In contrast, several functions have been attributed to UK114: endoribonuclease activity, p14.5 inhibitor of protein synthesis, activator of the μ-calpain protease. UK114 was also described as HRP12 (heat-responsive protein 12) involved in mRNA decay [38,39,40,41]. These multiple activities, which may need independent confirmation, are difficult to reconcile with the current knowledge on the Rid family. However, it cannot be ruled out that different members could be endowed with similar activity, but function in different processes [4]. Studies on *S. enterica* strains lacking RidA protein highlighted the interchangeable function of RidA proteins in preventing damage by 2AA. The inhibition of *S. enterica* IlvE by 2AA that is generated by the threonine/serine dehydratases IlvA or TdcB, two PLP-dependent enzymes, was assessed both in vitro and in vivo [3]. In an in vitro reconstituted system, the human UK114, as well as other homologs from an archaeon and from plants, proved to be as proficient as *S. enterica* YigF in preventing the dehydratase-dependent inhibition of IlvE [3]. Additionally, *S. enterica* YigF could be replaced by human UK114 and other homologs in an in vitro assay of inhibition of phosphoribosylamine formation [2], and human UK114 can complement the defective phenotype of an *mmf1*∆ of *S. cerevisiae* [33].

Here, we report the first specificity study of goat UK114 showing that it is indeed endowed with RidA activity. Both snake venom l-amino acid oxidase (LAAO) and pig kidney d-amino acid oxidase (DAAO) were used to generate the imino acid substrates for testing UK114 activity. Moreover, studies on the conformational stability of UK114 revealed an impressive robustness of this enzyme. The results provide a basis to explore the function of RidA proteins in mammals, and may also contribute to re-evaluating the antigenic property of UK114 in terms of cross-reactivity among pan-allergens belonging to the RidA protein family and in the studies on the relationship between allergy and tumors.

## 2. Results

### 2.1. Characterization of UK114 from Capra hircus (Goat)

**Figure 2:**
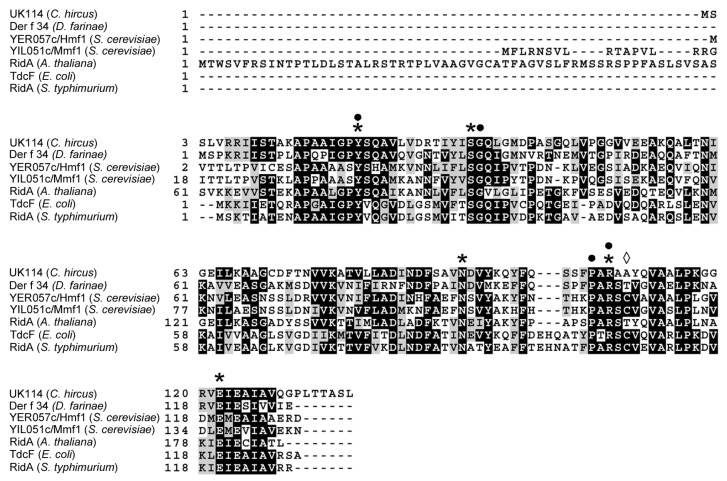
Sequence alignment of UK114 with RidA proteins. Amino acid sequences of members of the RidA family from plants, insects, yeast, and bacteria were aligned using Clustal Omega at EMBl-EBI and shaded using the Expasy Box Shade server. From top the amino acid sequences and their UniprotKB accession number are: *Capra hircus* UK114 (P80601), *Dermatophagoides farinae* Der f 34 (A0A1J1DL12), *S. cerevisiae* YER057c/*HMF1* (P40037), and YIL051c/*MMF1* (P40185), *A. thaliana* RidA (Q94JQ4), TdcF from *E. coli* (P0AGL2), and RidA from *Salmonella typhimurium* (Q7CP78). Similar residues are boxed in grey and identical residues are boxed in black. The stars indicate that the conserved residues of the active site and the diamond the residue that is not conserved with respect to the signature pattern of *E. coli* TdcF [6]. Closed circles: residues required for oligomerization [19,43].

Like most members of the Rid protein family, UK114 is a small protein of 137 amino acids (predicted mass 14,298.44) that assembles to form a homotrimer [42]. Remarkably, it lacks tryptophan and histidine residues. The only cysteine residue present is not engaged in the formation of disulfide bonds. UK114 shares the highest homology with the RidA proteins from sheep (*Ovis aries*) and from the domestic cow *Bos taurus* (99% and 98% identity, respectively), *Homo sapiens* (85%), *Rattus norvegicus* (82%), *Mus musculus* (81%), and rabbit (*Oryctolagus cuniculus*, 81%). The amino acid identity decreases to 76%, 65%, and 51% with the homologs from zebrafish (*Danio rerio*), *Caenorabditis elegans,* and *Drosophila melanogaster,* respectively. The amino acid identity with the RidA protein homolog from the model plant *Arabidopsis thaliana* is 42% and with Der f 34 from the house dust mite *Dermatophagoides farinae* is 47%. YIL051c/*MMF1* and YER057c/*HMF1*, the two paralogs from the baker’s yeast *Saccharomyces cerevisiae* share a slightly lower but significant identity with UK114 (42% and 38%, respectively). Because of the sequencing of many genomes, hundreds of sequences of Rid proteins from Eubacteria and Archaea are available and the extent of amino acid identity varies. The identity between UK114 and the RidA of *S. typhymurium* or of *E. coli* (TdcF), taken as the reference of the bacterial RidA, is 41%. The multiple alignment in Figure 2 shows the blocks of the most conserved amino acids in UK114 and other representatives of the RidA protein family. Extra N-terminal sequences are present in the mitochondrial RidA encoded by the yeast homolog YIL051c/*MMF1*, and in the RidA from *Arabidopsis thaliana*, which is a nuclear-encoded chloroplast enzyme, in agreement with the presence of import signals into the mitochondrion and into the chloroplast, respectively. A recent evolutionary analysis based on genomic data identified the signature pattern of conserved amino acids that are shared by the members of the RidA subfamily, whose reference protein is *E. coli* TdcF [6]. As shown in Figure 2, the Tyr17, Ser30, Asn88, Arg105, and Glu120 residues found in the active site of *E. coli* TdcF have an equivalent counterpart in residues Tyr21, Ser34, Asn93, Arg107, and Glu122 of UK114. TdcF Cys107, which is not conserved in all of the homologs, is replaced by Ala109 in UK114. Furthermore, amino acid residues that prevent the oligomeric assembly of monomeric UK114 in trimeric structures are the conserved residues Pro105, Arg107, Gly35, and Tyr21 of UK114 [19,43].

### 2.2. Expression and Purification of a Recombinant Goat UK114

A recombinant form of goat UK114 was produced in *E. coli*. The protein was isolated from cell extracts by Ni^++^-affinity chromatography, subjected to thrombin cleavage to remove the N-terminal His-tag, and then purified by gel filtration (see Materials and Methods). SDS-PAGE of the purified protein showed the presence of a single polypeptide of about 14 kDa (Figure S1a). Protein purity, as determined by liquid chromatography-mass spectrometry (LC-MS), was above 95% (Figure S1b). Analytical gel filtration on a calibrated Superdex 75 column showed that UK114 eluted in a single symmetrical peak with an apparent mass of about 36.4 kDa (Figure S2a). This value, which is lower than 43 kDa, is in good agreement with the assembly of UK114 into a compact homotrimer, as reported in a previously published study [44]. Native mass spectrometry (MS) confirmed the homotrimeric structure of UK114 yielding a molecular mass of 43736 Da although the monomeric form was also detectable due to protein denaturation during the electrospray process (Figure S2b). Protein sequencing was complete (100% coverage with high confidence) and is consistent with the expected sequence harboring three extra amino acids (GSH) when compared to the predicted UK114 sequence in protein database and four extra amino acids at the N-terminal (GSHM), with respect to the natural UK114 that lacks the initiator methionine and is *N*-acetylated on Ser2 [21]. No traces of miscleaved His-tag were detected (Table S1).

Secondary structure composition of UK114 in solution was estimated from the far-UV circular dichroism (CD) spectra recorded at 20 °C, and normalized in terms of mean residual ellipticity ([Ө]_MRW_) (Figure S3). Deconvolution of the spectrum results in the following composition: α-helix 18.7%, antiparallel β-sheets 20.2%, parallel β-sheets 5.6%, β-turn 18.6%, and random coil 33.6%. The secondary structure composition estimated from spectroscopic data is in good agreement with the crystallographic structure of UK114 (PDB entry 1nq3, [42]) that contains, out of the total 137 residues of each monomer, 31 amino acids that are located in α-helical regions (22.6%) and 42 in β-sheet regions (30%). Moreover, two out of the six strands characteristic of each monomer in the crystallographic structure are parallel, thus supporting the results that were obtained from the deconvolution.

### 2.3. C. hircus UK114 Is a Deiminase

**Figure 3:**
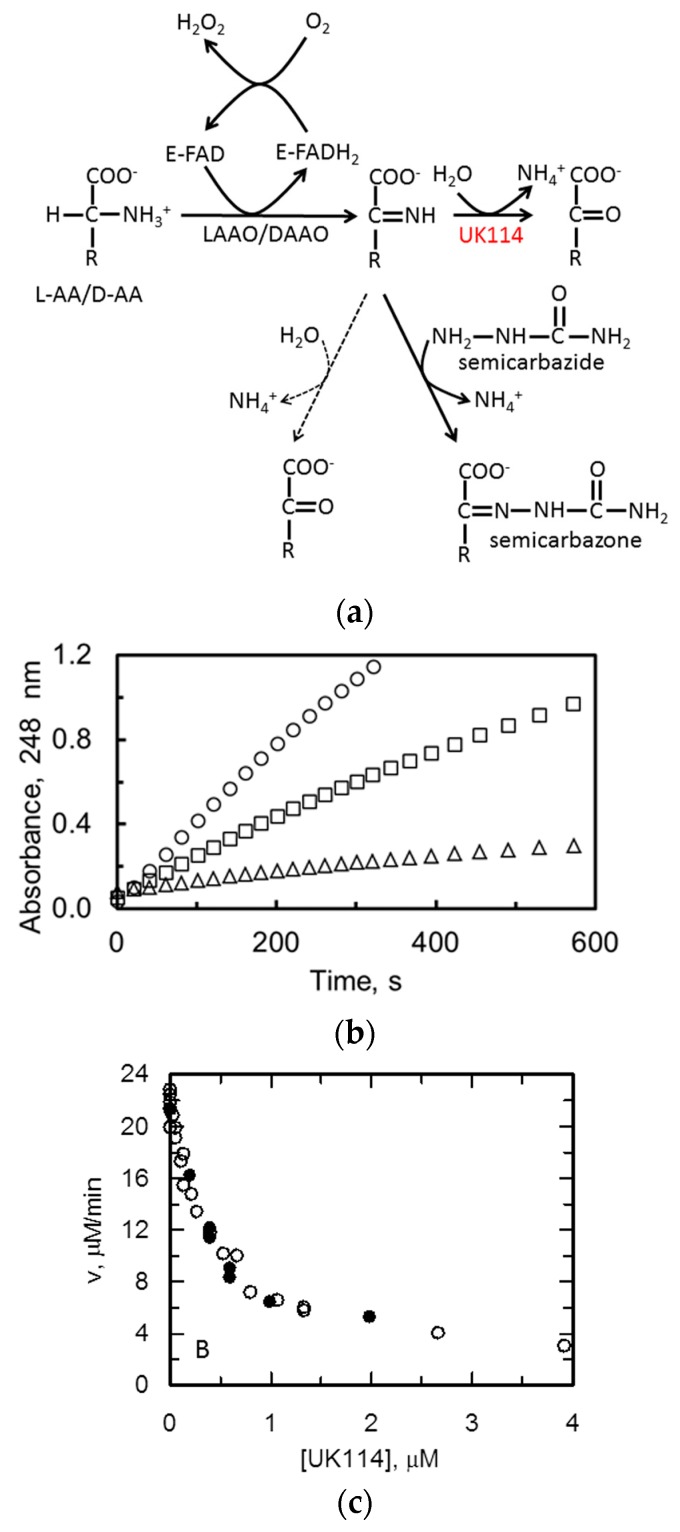
Deiminase activity of UK114. (**a**) Scheme of the assay used to monitor UK114 deiminase activity. Activity assays of the deiminase activity of UK114 were carried out essentially as described by [6] and derived from [11]. The method exploits the formation of semicarbazone from the imino acid released from l-amino acid oxidase (LAAO) reaction in the presence of l-amino acids, which is four orders of magnitude faster than semicarbazone formation from the corresponding keto acid at pH 8.7 [11]. Thus, the activity of UK114 can be measured from the decrease of the velocity of semicarbazone formation. The possibility to substitute d-amino acid oxidase (DAAO) and d-amino acids for LAAO and l-amino acids was also tested in this work. The dashed arrows indicate the spontaneous hydrolysis of the imino acid, which competes with semicarbazone formation (**b**) Time-course of absorbance changes at 248 nm monitoring the formation of semicarbazone from semicarbazide (10 mM) and the imino acid produced by LAAO (11 µg) and l-Leu (5 mM) in the absence (circles) or in the presence of UK114 (squares, 0.4 µM; triangles, 3.9 µM). The assays were carried out in 50 mM sodium pyrophosphate, pH 8.7, at 25 °C, in a final volume of 0.15 mL and included 1 μg of catalase as in Ref. [6]. (**c**) Scatter graph of initial velocity values of semicarbazone formation from l-Leu (5 mM) and LAAO (11 μg) in the presence of varying UK114 concentrations in 0.15 mL assays (open circles). The data from three independent experiments are shown along with those obtained in a similar experiment in which the imino acid was obtained from d-Leu and d-amino acid oxidase (closed circles). Error associated with measurements carried out under identical conditions was always less than 10%.

The enzymatic activity of the purified recombinant protein was tested by a previouslypublished assay, which used l-leucine (l-Leu) as the substrate of the LAAO reaction ([6], Figure 3). This assay measures the decrease of the rate of (non-enzymatic) semicarbazone formation from the 2-imino acid produced by snake venom LAAO due to hydrolysis of the imine catalyzed by UK114 (Figure 3a). The addition of increasing amounts of UK114 in the assay decreased the production of semicarbazone in a dose-dependent manner, which is in agreement with the hydrolysis of the imino acid catalyzed by UK114 and its consequent subtraction from the reaction with semicarbazide (Figure 3b). For this reason, and for simplicity, we shall refer to this UK114 activity as a deiminase reaction instead of enamine/imine deaminase.

An approximately linear relationship between the activity and the enzyme concentration was observed for UK114 concentrations up to 0.1 µM. Greater than 80% decrease of the rate of semicarbazone formation was observed with approximately 4 µM UK114 (Figure 3c).

In principle, the imino acid could be produced also by DAAO from the corresponding d-amino acid provided its hydrolysis to the corresponding α-keto acid, and ammonia does not occur prior to the release from the enzyme active site. As shown in Figure 3c, UK114 activity using the imino acid produced by DAAO from d-Leu was indistinguishable from that observed using LAAO and l-Leu. These results further demonstrate that UK114 is a RidA protein and also show that the substrate of UK114 is the α-imino acid, irrespective of the mode of its generation.

#### Specificity of UK114 toward Different Imino Acids

**Figure 4:**
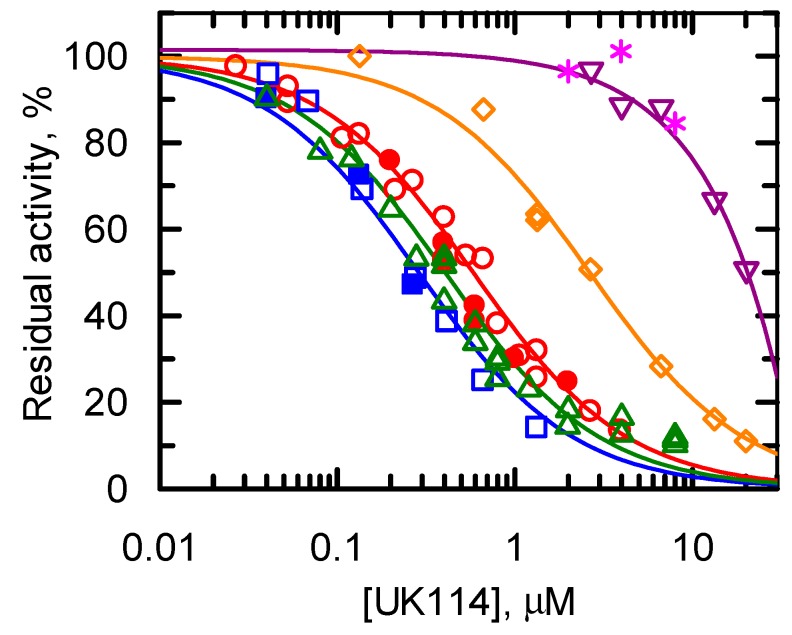
Specificity of UK114. l-Ala (open squares, blue), l-Leu (open circles, red), l-Met (open triangles, green), l-Gln (open diamonds, orange), l-Phe (inverted open triangles, purple), and l-His (stars, fuchsia, 5 mM each) were incubated with 10 mM semicarbazide in 50 mM sodium pyrophosphate buffer, pH 8.7, in the presence of catalase (10 μg/mL), LAAO (5–800 μg/mL), and varying UK114 concentrations at 25 °C. The results obtained using d-Ala (closed blue squares) and d-Leu (closed red circles) and DAAO (2–20 µg/mL) were indistinguishable from those that were obtained with the corresponding l-amino acid and LAAO. Thus, they were fitted together to Equation (1) obtaining the values of the concentration of UK114 that halves the velocity of semicarbazone formation shown in Table 1. The data obtained with l-Phe were fitted with a straight line. Due to the low effect of l-His, the analysis was limited and no data fitting was performed.

To extend the knowledge on the catalytic properties of goat UK114 in comparison with those of other RidA, we tested the enzyme activity on the α-imino acids that were generated in situ by LAAO and amino acids selected as representative of various classes (Figure 4, Table 1). UK114 was mostly active on the derivative of l-Ala, followed by l-Met and Leu. It was less active on l-Gln and poorly active on l-Phe and l-His (Figure 4, Table 1). The results obtained not only using d-Leu, but also d-Ala and DAAO are reported in Figure 4, and are indistinguishable from those obtained with the corresponding l-amino acids and LAAO. The curves that are shown in Figure 4 are the fit of the data to Equation (1), which allows the calculation of the concentration of UK114 that halves the initial velocity of semicarbazone formation measured in its absence (See Supplementary Materials). The values of the parameters and their standard errors are summarized in Table 1.

In summary, UK114 can use multiple substrates, but exhibits little or no activity on l-Phe and l-His, while it is active on imino acids from Ala, Leu, l-Met, and, to a lower extent, l-Gln, qualitatively paralleling *S. enterica* RidA specificity [6,15]. That the highest activity is obtained with the product of the oxidation of l- and d-Ala by LAAO and DAAO, respectively, is particularly interesting. Indeed, the 2-iminopyruvate (2-iminopropionate) is the tautomer of 2-aminoacrylate produced by both serine and cysteine dehydration, which is believed to be the RidA physiological substrate [2,3,5,35].

### 2.4. Study of the Conformational Stability of Goat UK114

**Figure 5:**
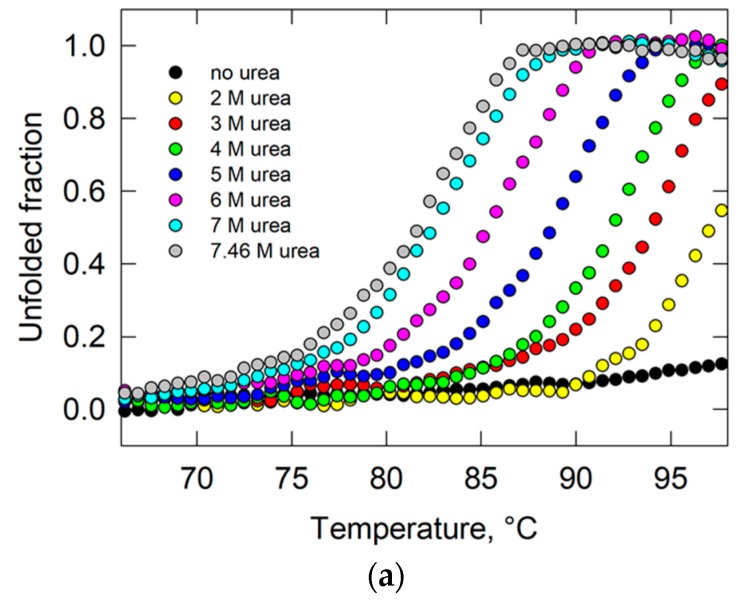

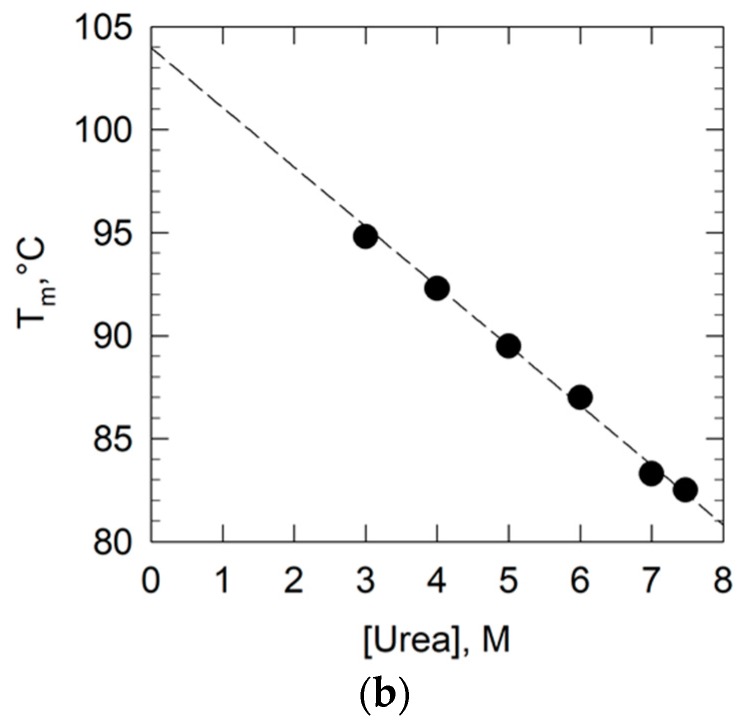
Thermal stability of UK114. (**a**) Temperature ramps in the absence or presence of urea at the indicated concentrations; the fraction of unfolded protein was determined from the changes in ellipticity at 220 nm. (**b**) Linear relation between the T_m_ derived from the data in panel (**a**) and urea concentrations.

To obtain information on the UK114 thermal stability, its unfolding was monitored by circular dichroism (CD) by measuring the change in ellipticity of the protein at 220 nm, which reports on the secondary structure content of the protein. An almost flat CD profile was obtained in the range of temperature from 20 to 98 °C, indicating that the protein conformation was not affected by the temperature increase (Figure 5). Also, other methods, such as Thermofluor analysis assays using Sypro Orange, gave similar results [45].

Since urea is known to facilitate unfolding, the CD experiments were repeated in the presence of different urea concentrations. At 2 M urea, protein unfolding started to occur, but only at high temperature (onset ≥ 90 °C) and the transition was not completed in the monitored temperature range. Starting from 3 M urea, the unfolding curves were fully described, allowing for the determination of melting temperatures (T_m_). At increasing concentrations of urea, the curves gradually translated toward relatively lower temperatures, but at 7.46 M urea the T_m_ was still high (82.5 °C, Figure 5). Notably, the values of T_m_ measured from the thermal unfolding curves were linearly related to the urea concentration (Figure 5b). Thus, the loss of cooperative interactions seems to be directly proportional to the urea concentration in the sample.

In addition, the completely unfolded protein was unable to refold, indicating that unfolding is an irreversible process for UK114 [46].

Finally, we tested the effect of perchloric acid on UK114 since this protein was first identified as a component of a perchloric acid-soluble extract of goat liver [21]. The perchloric acid-treated recombinant UK114 retained the activity and also the conformation of the untreated protein [47]. Also, freeze-drying, in the past used for the storage of the goat liver extract, did not affect the activity of the protein after reconstitution [46]. Overall, these results further highlight the extreme conformational stability of the protein.

### 2.5. Behavior of the Conformational Stability and Enzymatic Activity of UK114 Upon Long-Term Incubation at High Temperature

**Figure 6:**
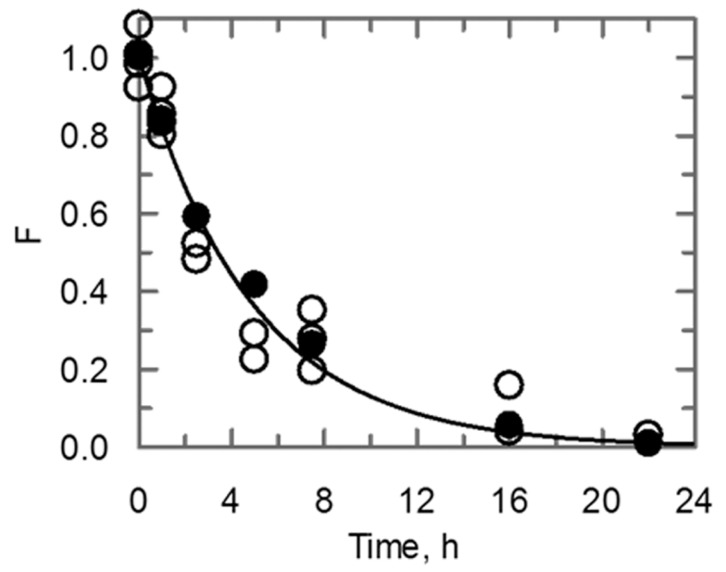
Time course of heat inactivation of UK114. Aliquots (120 µL) of UK114 (0.3 mg/mL, 20 µM) were set up in separate test tubes and incubated at 95 °C for the indicated times. Activity assays were carried out using different amounts of UK114 solution to ensure that the initial velocity values were calculated at a UK114 concentration where the relationship between initial velocity and UK114 concentration was linear. The fraction (F) of residual active protein was calculated from the difference between the initial velocity measured in the absence and in the presence of UK114 (open circles) at different times with respect to the value measured on a sample that had not been incubated at 95 °C, after normalization for the UK114 concentration in the assays. The circular dichroism (CD) spectrum of parallel samples was measured and the fraction (F) of residual folded protein was determined from the ellipticity at 220 nm (closed circles). The activity and CD data were fitted together to Equation (3), obtaining an apparent rate constant for unfolding and activity loss of 0.2 ± 0.02 h^−1^ and the expected initial value of F (F_0_) of 1.00 ± 0.03.

To further investigate the stability of UK114, its behavior was monitored during incubation at 95 °C for several hours. At different time intervals, protein conformation was analyzed by CD and RidA (deiminase) activity was assayed with l-Leu and LAAO. Both protein conformation and activity were gradually lost at 95 °C (Figure 6). After 3.5 h of incubation, the residual fraction of active/fully folded protein was about 50%. Notably, the extent of protein denaturation (measured by CD) and inactivation (measured through activity assay) were in excellent agreement and could be described as a single exponential process in which the protein irreversibly denatured with an apparent rate constant of 0.2 ± 0.02 h^−1^.

## 3. Discussion

In this work we defined that UK114 is a deiminase that exhibits properties that attribute it to the RidA subfamily, as also suggested by the presence of the conserved Arg107 (equivalent of Arg105 of other RidA) in the signature consensus pattern (Figure 2 and [6]). The specificity of UK114 toward various α-imino acids was compared to that reported for other RidA proteins. In the following, the amino acid is indicated instead of the imino acid for simplicity. The specificity study revealed a preference in the following order: Ala > Leu = l-Met > l-Gln. Little or no activity was detected using l-Phe and l-His. The data on l-Leu, l-Met, l-Gln, l-Phe, and l-His are similar to those that are reported for the RidA of *S. enterica* [6,15]. In other organisms, Rid2 and Rid3 proteins are active on l-Phe and l-His, but no homologs of these Rid subfamilies were detected in eukaryotes [15]. Interestingly, by generating the imino acids with l- and d-amino acid oxidases and the corresponding l- and d-amino acid under identical conditions, we showed—for the first time—that the highest specificity is indeed observed with 2-iminopyruvate (2-iminopropionate). This compound is the proposed physiological substrate of RidA proteins being the tautomer of the toxic 2-aminoacrylate generated by serine/threonine dehydratase and cysteine desulfhydrase [2,3,5]. It should be noted that—so far—the latter enzymes have been used to generate the RidA substrate, leaving some uncertainty about the nature of its actual substrate: the enamine or the imino acid. Therefore, the finding of activity with the product of the reaction of LAAO and DAAO and l- and d-Ala (or l- and d-Leu), respectively, clarifies that the actual RidA substrate is most likely the imino acid, rather than the corresponding enamine. In addition, our results provide a simple assay for testing the deiminase activity of Rid proteins, even with the proposed physiological substrate (2-iminopyruvate) with LAAO and DAAO that are commercially available.

The RidA protein UK114 is likely to have provided a solution to the phenomenon called metabolite damage, which is caused by accumulation of reactive enamines/imines, also in mammalian cells. This basic function appeared in early evolution (before the divergence of the three domains of life) and was maintained, an index of its important biological role. Since also PLP-dependent enzymes appeared early in evolution [48,49], it can be hypothesized that the selective pressure on RidA proteins was imposed by the co-presence of reactions catalyzed by PLP-dependent enzymes that generated toxic compounds, such as 2AA, and others that are sensitive to 2-AA [6]. The accumulation of 2AA was reported in cells devoid of RidA activity and 2AA stress is now recognized as an unbalanced metabolic condition that can cause several phenotypic defects. Thus, RidA proteins play an important function by contributing to the metabolic homeostasis on which cells rely on to survive. The fact that UK114 is maximally expressed in the liver, which is a crucial organ for amino acid metabolism, could be further explored by using (e.g.,) knock-out mice to understand the biological role of RidA proteins in mammals. Whether UK114 serves to hydrolyze imines produced by enzymes other than serine/threonine dehydratases or cysteine desulfhydrase, such as for example LAAO and DAAO, monoamine oxidases, or other amine oxidases, will need to be established to further clarify both its mechanistic features and its biological role in various organisms and tissues. The latter would be very interesting in the light of the known or proposed roles of these enzymes in fundamental processes and disease [50,51,52,53]. However, the feasibility of these studies will depend on the ability to set up robust activity assays, which will also allow for carrying out in depth mechanistic work.

Incidentally, RidA proteins (and—among them—UK114) may be used as mechanistic tools to establish whether l- and d-amino acid oxidases, and, perhaps, other amine oxidases actually release the imine or its hydrolysis takes place in the enzyme active site [54,55]. RidA has been indeed used to this purpose during the study of the type 2 iron-sulfur cluster-dependent l-serine dehydratase from *Legionella pneumophila* [56]. Furthermore, RidA proteins may find an application in biotechnological processes that exploit l- and/or d-amino acid oxidases in conjunction with PLP-dependent enzymes (e.g., deaminases, transaminases, and racemases) in order to avoid side reactions and/or biocatalyst inactivation that are caused by reactive imines [57].

In spite of the fact that human UK114 was annotated as a heat-responsive protein (HRSP12) (http://genome.ucsc.edu/), no data were available on its thermal stability. Interestingly, goat UK114 proved to be extremely heat resistant. Even at 7.46 M urea, the T_m_ was still high being about 82.5 °C. Thus, it would be of interest to explore the structural determinants that are responsible for the stabilization of this protein. Consistent with this notion, other harsh conditions, like treatment with perchloric acid or a cycle of freeze-drying/solubilization, did not affect stability and activity of the protein. No comparison could be made with other studies, since—to our knowledge—data on the thermal stability of RidA proteins are not available in the literature.

Goat UK114 has been reported to be endowed of remarkable antigenic properties [22,24,25]. Anti-UK114 antibodies were found to selectively label and exhibit complement-dependent cytotoxic activity on adeno-carcinoma cells (from colon, lung, breast), but not on normal cells, except for a small fraction of hepatocytes of fetal origin [23]. Moreover, UK114 is also related to Der f 34 that is able to bind IgE from asthmatic patients [37]. In this respect, it is worth noting that allergens are often very robust proteins. With the demonstration of the catalytic activity of UK114 and reference values for its specificity and conformational stability it will be possible to establish whether some of the effects of UK114 are related to its catalytic activity as a scavenger of toxic metabolic intermediates and/or its antigenic properties.

## 4. Materials and Methods

### 4.1. Construct for the Expression of Goat UK114 in E. coli

The recombinant plasmid for the expression of UK114 in *E. coli* was obtained by cloning the *Nde*I/*Xho*I-digested DNA fragment encoding goat UK114 into the corresponding sites of the pET15b expression vector (Novagen). This generates an in-frame fusion between the sequence encoding the N-terminal peptide MGSSHHHHHHSSGLVPR/GSH, comprising a 6xHis-tag and the cleavage site for thrombin (/) inside the sequence recognition site (underlined), and the CDS of UK114. PCR amplification was carried out using the primers UK-Nde-FOR (5′-AGCATATTCGACTGACATATG*TCGTCTTTGGTCAGAAGGAT*-3′) and UK-Xho-REV (5′-ATCGTCGGGCTCACTCGAG**CTA***GAGTGATGCTGTCGTGAGA*-3′), where the *Nde*I and *Xho*I sites are underlined, the coding sequence of UK114 is in italic and the stop codon is in bold, the Phusion^®^ Hot Start High-Fidelity DNA Polymerase (New England Biolabs, NEB), and a previously described plasmid harboring the cDNA of the goat UK114 as a template [21]. The purified PCR product of about 450 bp and pET-15b were double-digested with *Nde*I and *Xho*I (NEB). The gel-purified PCR band and the linearized vector were ligated (Quick Ligation Kit, NEB) and transformed into *E. coli* DH5α cells. The recombinant plasmid, named pET-15b-His-UK, was sequenced (BMR Genomics, Padova, Italy) to verify the absence of mutations in the cloned sequence and the correct fusion. pET-15b-His-UK was used to transform *E. coli* Rosetta (DE3) strain (Novagen) for protein production.

### 4.2. Protein Production and Purification

*E. coli* Rosetta (DE3) strain harboring pET-15b-His-UK was grown in 750 mL of Luria-Bertani medium [0.5% (*w*/*v*) yeast extract, 1% (*w*/*v*) peptone, 0.1% (*w*/*v*) glucose, 0.5% (*w*/*v*) NaCl] with 100 µg/mL ampicillin, and 20 µg/mL chloramphenicol at 37 °C until the optical density at 600 nm (OD_600_) reached 0.6. Isopropyl β-d-1-thiogalactopyranoside (IPTG) was added to a final concentration of 0.5 mM. After 4 h at 37 °C, the culture was cooled in an ice bath and cells were harvested by centrifugation at 4 °C. The pellet (9 g of wet weight of cells) was suspended in Lysis buffer (50 mM Na phosphate, pH 7.4, 300 mM NaCl, 0.5 mM MgCl_2_, containing 1 mM phenylmethylsulfonyl fluoride (PMSF), Complete protease inhibitor cocktail (Roche Applied Sciences, Penzberg, Germany) and DNaseI (Qiagen, Hilden, Germany) in a 10:1 ratio of buffer (mL)/gr of cell wet weight. After cell breakage with a French press, the crude extract was centrifuged at 15,000× *g* for 40 min at 4 °C in a Sorval RC5 centrifuge and the supernatant was applied to a Nickel-Sepharose column (HisPrep FF16/10, GE Healthcare Life Sciences, Pittsburgh, PA, USA, 20 mL), equilibrated with 50 mM Na phosphate buffer, pH 7.4, 300 mM NaCl, 30 mM imidazole connected to a FPLC system. The column was extensively washed with the equilibration buffer and His-tagged UK114 was eluted with a linear 30 mM–1 M imidazole gradient in 50 mM Na phosphate buffer, pH 7.4, 300 mM NaCl (60 mL; 3 column volumes), monitoring the absorbance at 280 nm. After SDS-PAGE analysis, the fractions containing the recombinant protein were pooled and dialyzed for 16 h at 4 °C against 10 mM Tris-HCl, pH 7.4, 300 mM NaCl. To remove the tag, CaCl_2_, and thrombin (GE Healthcare) were added to the dialyzed fraction at a concentration of 2 mM and 5 U/mg of tagged UK114, respectively, and incubated for 2 h at 27 °C. The fully digested protein was concentrated to a final volume of 10 mL in Amicon Ultra centrifugal filter devices (cut off 10 kDa, 4 mL, Millipore) and applied to a Superdex 75 (26/60) gel filtration column (GE Healthcare) previously equilibrated with 0.9% NaCl. The fractions containing UK114 were pooled after SDS-PAGE analysis. The removal of the tag by thrombin leaves three extra amino acids (GSH) at the protein N-terminus as compared to the predicted UK114 protein sequence (UniProt P80601). The identity of the fusion protein with the expected amino acid sequence was assessed by MS analysis of the purified protein (see Section 4.6). This procedure yields about 200 mg homogeneous UK114 from 9 g of starting *E. coli* wet paste.

### 4.3. Analytical Gel Filtration

To estimate the native molecular mass of recombinant UK114 in solution, the Superdex 75 (10/30) analytical gel filtration (GE Healthcare) equilibrated with 0.9% NaCl was used. The column was calibrated with standard proteins under the same conditions (BSA dimer, 132 kDa; BSA monomer, 66 kDa; carbonic anhydrase, 29 kDa; cytochrome c, 12.3 kDa). The calculated molecular mass of the recombinant UK114 monomer was 14,759.71 Da.

### 4.4. Protein Purity Determination by Nanoscale Liquid Chromatography—Mass Spectrometry (LC-MS)

The analyses were performed on an analytical platform that was composed of a UltiMate3000 nano-flow LC system connected to an LTQ-Orbitrap XL mass spectrometer through a nanoscale electrospray ionization source (NSI) with a stainless-steel emitter (5 cm length, O.D. 150 µm, I.D. 30 µm, Thermo Fisher Scientific, Rodano, MI, Italy) assembled on a Finnigan NSI-1 dynamic probe. A 0.3 µL aliquot of UK114 protein solution (1 mg/mL in water) was picked up and transported to a PepSwift monolithic nano column (100 µm × 25 cm PS-DVB, Thermo Fisher Scientific, Rodano, MI, Italy). Elution was performed at a flow rate of 0.85 µL/min using 0.1% aqueous formic acid as mobile phase A and acetonitrile containing 0.1% formic acid as mobile phase B. The binary gradient program started with a 5 min isocratic flow at 10% solvent B, followed by a linear gradient of up to 50% B in 20 min; a faster linear ramp up to 95% B in 5 min was followed by an isocratic flow at 95% B for additional 3 min to wash the column before restoring the initial conditions and keep them for additional 4 min to prepare the column for the next injection. Sample nebulization was provided by a 1.7 kV voltage without nebulizing gas. A capillary temperature of 220 °C was applied to the entrance of the mass spectrometer to promote solvent evaporation. Positive ion mode mass spectra were acquired by the Orbitrap analyzer in a range between 600 *m*/*z* and 2000 *m*/*z*, setting a resolution of 100,000 (FWHM at 400 *m*/*z*), and activating the profile mode feature. A target of 5 × 10^6^ ions per scan and a maximum filling time of 500 ms were set to allow optimal signal intensity. A list of 20 identified background ions was included in the lock mass feature for real time mass calibration [58]. Instrument control and spectra analysis were provided by the software Xcalibur 2.0.7 and Chromeleon Xpress 6.80 (Thermo Fisher Scientific, Waltham, MA, USA).

### 4.5. Protein Conformational Integrity by Nanoscale Electrospray Mass Spectrometry (NSI-MS)

The analyses were performed under native conditions [59] by an automated loop injection method, which was developed on the analytical platform described above. A 1 µL aliquot of purified UK114 (1 mg/mL in 50 mM ammonium formate) was picked up and transported directly to the NSI source with no column in between. The mobile phase for sample transport was 10 mM ammonium formate at a flow rate of 0.3 µL/min. Sample nebulization was provided by a 1.5 kV voltage without nebulizing gas. A capillary temperature of 200 °C was applied to the entrance of mass spectrometer to promote solvent evaporation. Positive ion mode mass spectra were acquired by the Orbitrap analyzer in a range between 800 *m*/*z* and 4000 *m*/*z,* with the same settings being reported at Section 4.4. Mass spectra deconvolution was performed using the Xtract function of Xcalibur 2.0.7.

### 4.6. Protein Sequencing by Nanoscale Liquid Chromatography Tandem Mass Spectrometry (LC-NSI-MS/MS)

Protein sequencing was done as described in [60] with minor modifications concerning data analysis. No post-translational modifications were allowed and the protein database was built to include the predicted amino acid sequences of the His-tagged and of the thrombin-cleaved recombinant UK114. Such sequences were obtained by translating the nucleotide sequence of the DNA construct encoding the recombinant goat UK114 (see Section 4.1). The sequences MGSSHHHHHHSSGLVPRGSH or GSH preceded the N-terminal methionine of the goat UK114 predicted amino acid sequence (accession number UniprotKB P80601) in order to match the sequences of the His-tagged or thrombin-cleaved fusion UK114 protein, respectively.

### 4.7. Activity Assays of UK114 Deiminase Activity

The deiminase activity of UK114 was determined by measuring the decrease of velocity of semicarbazone formation from various α-imino acids generated in situ by l-amino acid oxidase (LAAO) from the corresponding l-amino acid (Figure 3a). Reaction conditions were similar to those that were described by [6] in order to allow for direct data comparison. Briefly, assay mixtures contained the l-amino acid (5 mM), neutralized semicarbazide (10 mM), catalase (10 µg/mL), snake venom LAAO (5–800 µg/mL, Sigma-Aldrich, Merck KGaA, Darmstadt, Germany), and varying amounts of UK114 in 50 mM sodium pyrophosphate buffer, pH 8.7. The reaction was started by either addition of the l-amino acid or LAAO and was monitored spectrophotometrically in a HP 8452A diode array spectrophotometer (Agilent Technologies, Santa Clara, CA, USA) by collecting spectra every 5–20 s, depending on the experiments. In the experiments done with different amino acids, the concentration of l-amino acid oxidase (5–800 µg/mL) was selected in order to obtain a similar initial velocity of semicarbazone formation in the absence of UK114 of 22–25 µM/min as monitored at 25 °C at 248 nm (ɛ_248_, 10.3 mM^−1^ cm^−1^). Again, the conditions were chosen to reproduce those described in [6] for better comparison of the results. Reactions (0.15–1 mL) were set up in quartz cuvettes (1 cm light path).

LAAO activity was also measured by monitoring the initial velocity of hydrogen peroxide production in a coupled assay with horse radish peroxidase (HRP) and o-dianisidine, as previously described for DAAO assays [61]. The assays contained the given amino acid (5 mM or varying concentrations for determination of the steady-state kinetic parameters *V*_max_ and *K*_m_), o-dianisidine (0.4 mM), HRP (10 µg/mL) and varying concentrations of LAAO (5–800 µg/mL). The increase of absorbance at 436 nm was monitored. An extinction coefficient of 11.6 mM^−1^ cm^−1^ was applied.

Under these conditions, by comparing the initial velocity of hydrogen peroxide formation with that of semicarbazone formation, 50–60 percent of the imino acid appears to be trapped as the semicarbazone (Figure S4 and Table S2). This result is in agreement with the finding that the semicarbazide concentration yielding 50% of the expected semicarbazone was 11.6 mM at pH 8.7, where the imino acid stability is high (*k*_hydrolysis_ 1.8 min^−1^ vs. 6 min^−1^ at pH 7.7), as reported in the early work by Hafner and Wellner [11], on which this assay is based. However, semicarbazide concentration was kept constant at 10 mM in order to allow direct comparison with data from other laboratories and to avoid inhibition of LAAO that is observed at higher concentrations. For the same reason, l-Leu concentration was kept constant at 5 mM, although it appears to inhibit LAAO (Figure S4).

Control experiments showed that UK114 at concentrations up to 20 μM did not interfere with the initial velocity of hydrogen peroxide production from l-Leu by LAAO.

The possibility to generate 2-imino acids from d-amino acids and pig kidney d-amino acid oxidase was tested by setting up activity assays for the measurement of the initial velocity of oxidation of selected d-amino acids (d-Leu, d-Ala, d-Ser, d-Thr, and d-Lys) as H_2_O_2_ production in the HRP-coupled assay and as semicarbazone formation. Assay conditions were identical to those described for l-amino acids and LAAO. DAAO was purified from pig kidneys, as previously described [62]. Semicarbazone formation was observed using d-Ala, d-Leu, but not d-Ser, d-Thr, or d-Lys, indicating that either semicarbazide cannot react with the imino acid that is formed from the latter three amino acids or (more likely) their hydrolysis takes place prior to release from DAAO active site [54]. Also, with d-Ala and d-Leu and DAAO, the amount of imino acid that is trapped by semicarbazide is approximately half that of hydrogen peroxide measured in the coupled assay with HRP.

To obtain estimates of the specificity of UK114 with respect to the hydrolysis of imines derived from the different amino acids, the initial velocity values of semicarbazone formation measured in the presence of varying concentrations of UK114 were fitted to Equation (1), which was derived as described in Supplementary Materials. In Equation (1), v_0_ is the initial velocity of semicarbazone formation measured in the absence of UK114; v is the initial velocity measured in the presence of a given UK114 concentration ([UK114]); and, K_50_ is the UK114 concentration that leads to measuring an initial velocity of semicarbazone formation (v) that is half that measured in the absence of UK114 (v_0_). The Grafit software (Erythacus Software Ltd., Horley, Surrey, UK) was used for data fitting to Equation (1).

(1) $$\frac{v}{v_{o}}=\frac{1}{1+\frac{\left[\mathrm{UK}114\right]}{K_{50}}}$$

### 4.8. Circular Dichroism

Circular dichroism (CD) studies were carried out on a J-810 spectropolarimeter (JASCO Europe, Cremella, Italy) equipped with a Peltier system for temperature control. All of the measurements were performed in a 0.1 cm path length cuvette at 0.2 mg/mL protein concentration in physiologic saline solution (0.9% NaCl) containing urea when indicated. Far-UV spectra were recorded from 195 to 260 nm at 20 °C, and normalized in terms of mean residual ellipticity ([Ө]_MRW_) [63]. Estimates of the secondary structure composition were obtained by means of CDNN software version 2.1 (http://bioinformatik.biochemtech.uni-halle.de/cdnn/ Copyright Gerald Böhm, Institut für Biotechnologie, Martin-Luther Universität Halle-Wittenberg) [64]. Temperature ramps were recorded from 20 to 98 °C at a heating rate of 1 °C/min, while continuously monitoring the CD signal at 220 nm. T_m_ values were determined as the maximum of the first derivative of the unfolding profiles.

### 4.9. Heat Inactivation of UK114 and Data Analysis

A UK114 solution (20 µM, 0.3 mg/mL) in physiological saline solution (0.9% NaCl) was divided into 120 µL aliquots in 500 µl-PCR tubes and incubated for different times at 95 °C (zero, 1, 2.5, 5, 7.5, 10, 16, and 22 h) in a Eppendorf 5331 MasterCycler Gradient Thermal Cycler (Eppendorf, Hamburg, Germany) with a hot lid to prevent evaporation. At different times, two aliquots was withdrawn, cooled, and briefly centrifuged to collect the sample at the bottom of the tube. After mixing, the sample was centrifuged again. No protein precipitation was detected. The CD spectra and the residual activity were measured on parallel samples.

CD-derived data were analyzed using a two-state mechanism. Unfolding curves for the N ↔ D transition were normalized to the apparent fraction of the folded (F) using Equation (2)

F = (Y − Y_D_)/(Y_N_ − Y_D_) (2)

In the equation Y is the observed variable parameter (CD signal at 220 nm) and Y_N_ and Y_D_ are the values that are characteristic of the native and fully unfolded conformations, respectively. To determine the extent of UK114 inactivation, activity assays were carried out using l-Leu, LAAO, and different amounts of UK114 solution to ensure that the initial velocity values at any given time were calculated at a UK114 concentration where the relationship between initial velocity and UK114 concentration is linear. After normalization for the UK concentration used, the fraction of residual activity (F) was calculated by dividing the measured velocity by the initial reaction velocity of a sample that had not been heat-treated.

CD and activity data were fitted together to Equation (3), describing a single exponential process, where F_0_ is expected to be equal to 1, *k* is the apparent rate constant for unfolding or inactivation, and *t* is the time in hours.

F = F_0_·e^−*kt*^ (3)

### 4.10. Treatments of UK114 with Perchloric Acid and with a Freeze-Drying-Reconstitutionprocess

UK114 (0.6 mg/mL) was diluted two-fold with 2 M perchloric acid. Solid sodium acetate was added to bring the pH to 4.1. Then the sample was incubated at 4 °C for 16 h. After dialysis against a volume of PBS (Phosphate buffered saline: 0.0033 M NaH_2_PO_4_, 0.0067 M Na_2_HPO_4_, pH 7.4, and 7.4 g/L NaCl) equal to 100× the sample volume for 24 h at 4 °C with one buffer change, the samples were filtered through sterile Millex filter units (pore size 0.22 µm, Millipore, Darmstadt, Germany) and aliquots were frozen in liquid nitrogen and stored at −20 °C until the CD spectrum and activity were measured.

Four hundred microliters of a 0.3 mg/mL UK114 solution in physiological solution or in PBS, pH 7.4, were frozen at −80 °C and lyophilized in Alpha 2–4 LD freeze-dryer (Christ, Osterode am Harz, Germany). After reconstitution in the initial volume with H_2_O, the enzymatic activity of UK114 was tested.

## Figures and Tables

**Table 1:** Specificity of UK114 ^1^.

Amino Acid	K_50_, μM	100/K_50_, μM^−1^
Ala	0.29 ± 0.02	344 ± 24
Leu	0.58 ± 0.03	172 ± 9
l-Met	0.41 ± 0.03	208 ± 17
l-Gln	2.56 ± 0.11	39 ± 2
l-Phe	ND	2.5 ± 0.2
l-His	ND	ND

^1^ Data of Figure 4 were fitted to Equation (1) to obtain the indicated values of K_50_, i.e., the concentration of UK114 that leads to measuring an initial velocity equal to half that measured in its absence. The data obtained with l- and d-Ala and with l- and d-Leu with the corresponding amino acid oxidases were fitted together. The K_50_ values for the activity in the presence of the imino acid derived from l-Phe and l-His could not be determined (ND) due to their low effect, even at high UK114 concentrations. The data obtained with l-Phe were fitted to a straight line. From the calculation of the limit for [UK114] → 0 of the first derivative of Equation (1) (100/K_50_), one can obtain an estimate of the percent decrease of rate of semicarbazone formation caused by 1 μM UK114, which could be converted into an apparent turnover number by taking into account the initial velocity value measured in the absence of UK114. For d-Phe, this value corresponds to the slope obtained from the fit of the data to a straight line.

## Supplementary Materials

Supplementary materials can be found at http://www.mdpi.com/1422-0067/19/4/945/s1.

## Abbreviations

Rid	Reactive intermediate deaminase
PLP	Pyridoxal 5′-phosphate
2AA	2-aminoacrylate
MS	Mass spectrometry
CD	Circular dichroism
LAAO	l-amino acid oxidase
DAAO	d-amino acid oxidase
