# Supplementary material for: Imine Deaminase Activity and Conformational Stability of UK114, the Mammalian Member of the Rid Protein Family Active in Amino Acid Metabolism
Source: Int J Mol Sci. 2018 Mar 22;19(4):945. doi: 10.3390/ijms19040945 (PMC5979572; doi:10.3390/ijms19040945)
Supplement: Supplementary file 1 [file ijms-19-00945-s001.pdf]

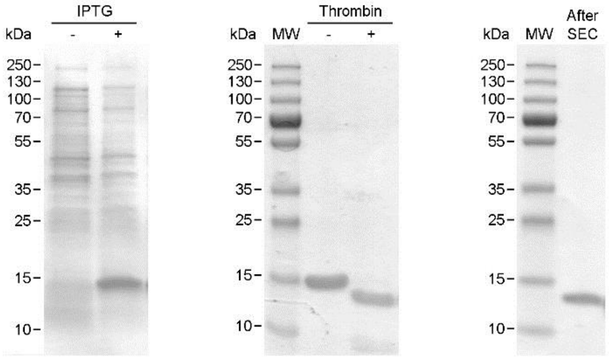

(**a**)

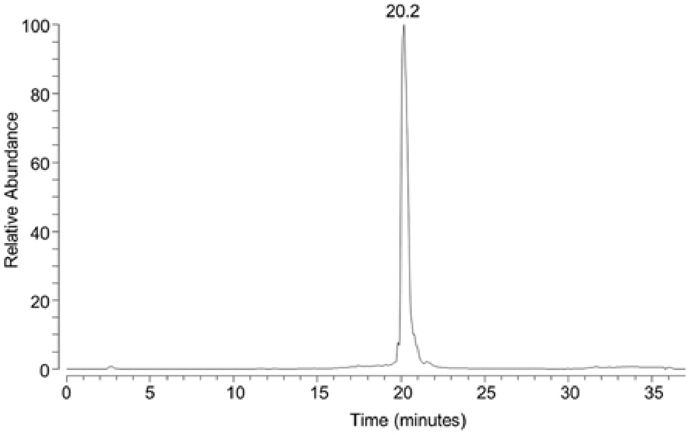

(**b**)

Figure S1. Production and purification of UK114. (**a**) SDS-PAGE analysis of cellular extracts from uninduced and induced *E. coli* cultures (left panel), thrombin cleavage of the His-Tag UK114 after Ni^{++}-affinity chromatography (center panel) and gel filtration of the digested protein (right panel). (**b**) Total ion current (LC-MS) trace of recombinant goat UK114. The protein purity is above 95%.

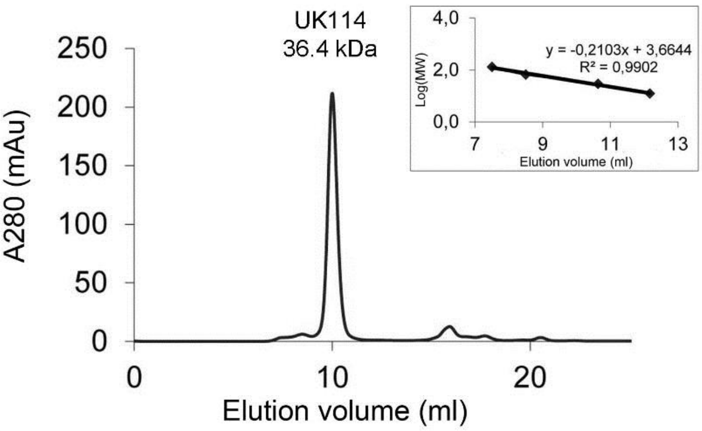

(**a**)

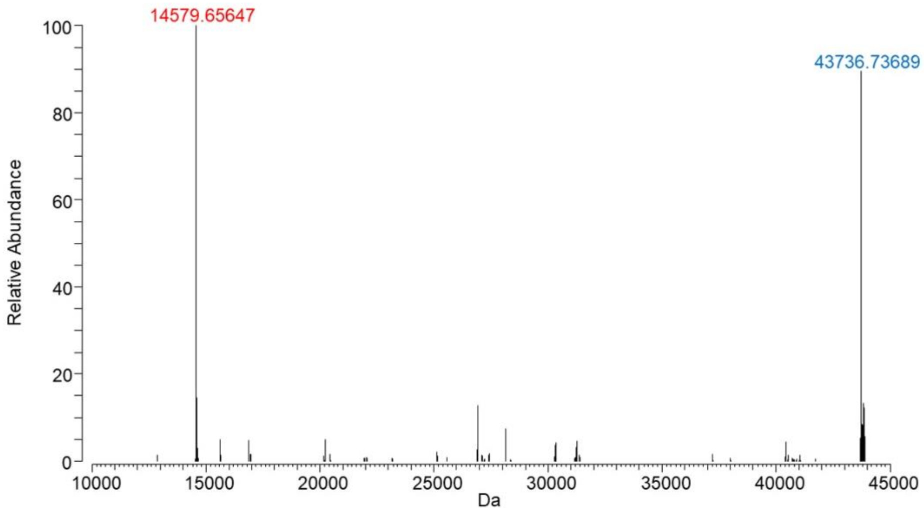

(**b**)

Figure S2. Recombinant UK114 is a homotrimer. (**a**) Analytical gel filtration. (**b**) Deconvoluted mass spectrum of goat UK114 analyzed under native conditions. The monomer mass is red, homotrimer mass is blue.

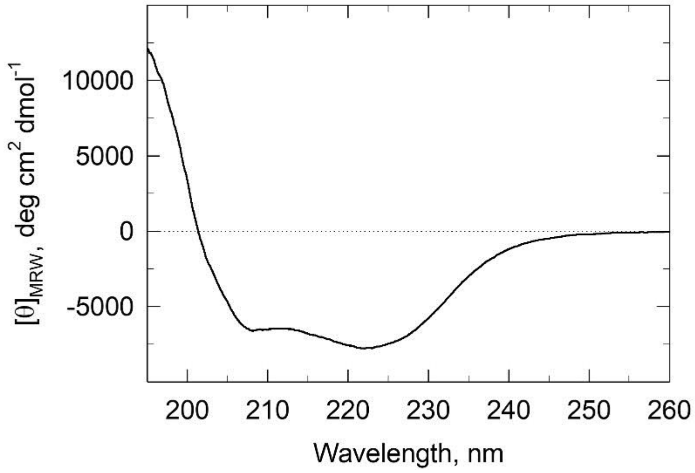

Figure S3. Far-UV CD spectrum of UK114. Spectrum of UK114 0.3 mg/mL in physiological saline solution (0.9% NaCl) was acquired a 20 °C in a 0.1 cm path length cuvette and normalized as Mean Residual Ellipticity ($[\Theta]_{MRW}$).

Table S1. SEQUEST output of the recombinant UK114 sequencing.

Sequence Position	Peptide Sequence	Xcorr	SpScore	Charge	*m/z*, Da	Delta Mass, PPM	RT, min
1–10	GSHMSSLVRR	2.94	479.02	2	565.2977	−1.31	4.03
11–16	IISTAK	1.39	221.06	2	316.70242	−0.33	2.56
17–32	APAAIGPYSQAVLVDR	5.79	916.05	2	814.4425	−1.58	6.53
33–59	TIYISGQLGMDPASGQLVPGGVVEEAK	6.13	834.15	3	906.13715	3.88	12.01
60–70	QALTNIGEILK	3.14	534.15	2	600.3526	−1.29	6.04
71–81	AAGCDFTNVVK	3.36	588.64	2	562.77295	−1.7	4.25
82–100	ATVLLADINDFSAVNDVYK	6.97	1702.5	2	1034.533	−0.56	15.01
101–110	QYFQSSFPAR	3.27	655.32	2	615.79797	−1.27	6.01
111–120	AAYQVAALPK	3.09	806.92	2	516.29712	−1.44	5.08
121–140	GGRVEIEAIAVQGPLTTASL	5.73	473.07	2	856.477	−0.84	14.02

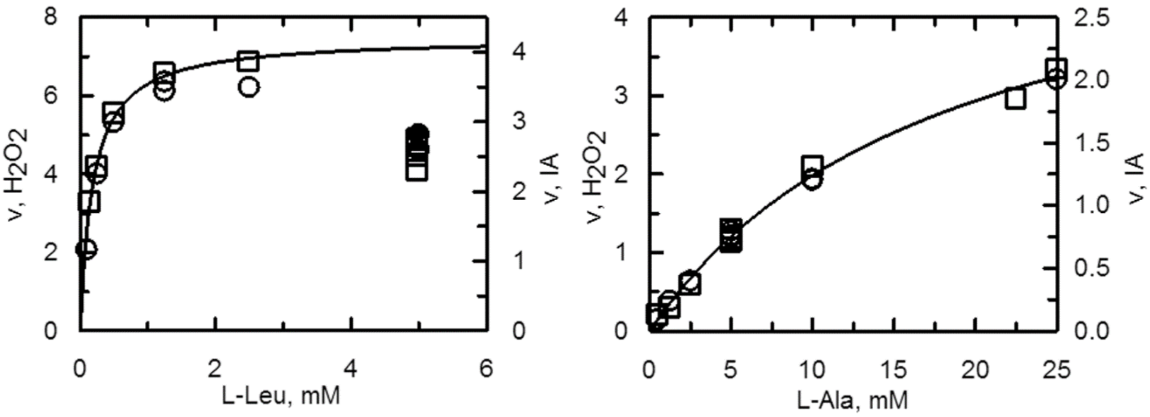

Figure S4. Determination of V_{max} and K_m for L-Leu (**left**) and L-Ala (**right**) for the reaction of LAAO as monitored from H_2O_2 production (v, H_2O_2; circles, left vertical axis) or semicarbazone formation from the imino acid (v, IA, squares, right vertical axis). The curves are the fit of the initial velocity data obtained by monitoring semicarbazone formation to the Michaelis Menten equation ($v = V_{max}*S/(K_m + S)$, with: v, initial reaction velocity; V_{max}, maximum velocity; K_m, Michaelis-Menten constant). The initial velocity is expressed as mM H_2O_2 or semicarbazone formed per minute per mg LAAO. The steady-state kinetic parameters calculated for the reactions are summarized in Table S2.

Table S2. Comparison of the steady-state kinetic parameters maximum velocity (V_{max}) and Km measured with LAAO in the presence of L-Leu and L-Ala obtained by monitoring hydrogen peroxide or imino acid (IA) formation.

**Amino Acid**	**H_2O_2 Formation Via Coupling with HRP Reaction**		**Imino Acid Formation Via Semicarbazone Formation**		
	V_{max}, $mM{\cdot}min^{-1}{\cdot}mg^{-1}$	K_m, mM	V_{max}, $mM{\cdot}min^{-1}{\cdot}mg^{-1}$	K_m, mM	V_{IA}/V_{H2O2}
L-Leu	7.1 ± 0.3	0.2 ± 0.03	4.2 ± 0.06	0.190 ± 0.01	0.59
L-Ala	5.5 ± 0.2	17.8 ± 1.0	3.5 ± 0.25	18.4 ± 2.4	0.63

Derivation of Equation (1)

$$\frac{v}{v_0} = \frac{1}{1 + \frac{[UK114]}{K_{50}}}$$

The deiminase activity of UK114 is measured as the decrease of initial velocity of formation of semicarbazone from the imino acid produced by L- or D-amino acid oxidase (Supplementary Figure S4). Thus, the system consists of two parallel competing pseudo-first order reactions: A → B, which occurs with an apparent rate constant k_B, and A → C, which takes place with an apparent rate constant k_C. A is the imino acid formed by L- or D-amino acid oxidase; B is the semicarbazone formed upon reaction of the imino acid with semicarbazide.

Since the initial velocity of the reaction is measured, semicarbazide concentration can be considered constant. Thus, the velocity of semicarbazone formation ($v_B = k_1*[A]*[semicarbazide]$) is a pseudo-first order reaction ($v_B = k_B*[A]$, with $k_B = k_1*[semicarbazide]$). Also the UK114-catalysed hydrolysis of the imino acid (A) into the corresponding keto acid and ammonia is a pseudo-first order reaction with $k_C = k_2*[UK114]$, with $k_2 = (k_{cat}*[A])/(K_m + [A])$, i.e., the velocity predicted by the Michaelis-Menten equation with k_{cat} and K_m being the turnover number and the K_m of UK114 with the given imino acid, respectively. Since the imino acid is generated in situ by the L- or D-amino acid reaction, at the same rate in all assays, and the initial velocities are measured, k_C can also be considered constant.

For two parallel competing (pseudo)-first order reactions, the concentrations of B and C can be written as in [1]:

$$[B] = [Ao] * \frac{k_B}{k_B+k_C} * \left[1 - e^{-(k_B+k_C)*t}\right] \text{ and } [C] = [Ao] * \frac{k_C}{k_B+k_C} * \left[1 - e^{-(k_B+k_C)*t}\right]$$

Thus, at any given time:

$$\frac{[B]}{[C]} = \frac{k_B}{k_C} \text{ and also: } \frac{v_B}{v_C} = \frac{k_B}{k_C}$$

Thus, rearranging: $v_C = v_B * \frac{k_C}{k_B}$

Since v_C is the difference between the velocity measured in the absence of UK114 (v_0) and the velocity of semicarbazone formation measured in its presence (v_B), the expression becomes:

$$v_0 - v_B = v_B * \frac{k_C}{k_B}$$

Thus: $v_B * \left(1 + \frac{k_C}{k_B}\right) = v_0$ and $\frac{v_B}{v_0} = \frac{1}{\left(1+\frac{k_C}{k_B}\right)}$

With $k_C = k_2$*[UK114] the expression of the % relative velocity (v) measured in the presence of UK114 becomes:

$$v = \frac{100}{\left(1 + \frac{[UK114]}{\frac{k_B}{k_2}}\right)}$$

By defining the concentration of UK114 that leads to measuring half the velocity of semicarbazone formation as $K_{50} = \frac{k_B}{k_2}$, one obtains Equation (1):

$$v = \frac{100}{\left(1 + \frac{[UK114]}{K_{50}}\right)}$$

With $k_2 = \frac{k_{cat}*[A]}{K_m+[A]}$, K_{50} is related to the specificity of UK114 for the different imino acids.

It should be noted that the curve relating the residual activity (in percent) and the UK114 concentration did not change when the initial velocity of formation of the imino acid (in the absence of UK114) was half that routinely used in these experiments. Thus, it appears that, under the assay conditions, UK114 works under k_{cat}/K_m conditions, i.e., its reaction velocity is linearly dependent upon substrate concentration and K_{50} relates to the catalytic efficiency of UK114 with the given imino acid.

The initial part of the curve relating residual activity to UK114 concentration is linear. By calculating the limit for [UK114] → 0 of the first derivative of the expression of v as a function of [UK114] one obtains the slope of the linear part of the curve, which corresponds to the apparent turnover number of UK114 with a given imino acid once it is multiplied by the initial velocity measured in the absence of UK114 expressed as μM semicarbazone formed/min

$$\lim_{UK114 \to 0} \frac{dv}{d[UK114]} = -\frac{100}{K_{50}}$$

Reference:

1. Fersht, A. *Structure and Mechanism in Protein Science*; W H Freeman: New York, NY, USA, 1984.
